# Effect and Safety of Shihogyejitang for Drug Resistant Childhood Epilepsy

**DOI:** 10.1155/2016/3410213

**Published:** 2016-03-16

**Authors:** Jinsoo Lee, Kwanghyun Son, Gwiseo Hwang, Moonju Kim

**Affiliations:** ^1^Department of Pediatric Neurology of Korean Medicine, I-Tomato Hospital, Seoul, Republic of Korea; ^2^College of Korean Medicine, Gachon University, Seongnam, Republic of Korea

## Abstract

*Objective*. Herbal medicine has been widely used to treat drug resistant epilepsy. Shihogyejitang (SGT) has been commonly used to treat epilepsy. We investigated the effect and safety of SGT in children with drug resistant epilepsy.* Design*. We reviewed medical records of 54 patients with epilepsy, who failed to respond to at least two antiepileptic drugs and have been treated with SGT between April 2006 and June 2014 at the Department of Pediatric Neurology, I-Tomato Hospital, Korea. Effect was measured by the response rate, seizure-free rate, and retention rate at six months. We also checked adverse events, change in antiepileptic drugs use, and the variables related to the outcome.* Results*. Intent-to-treat analysis showed that, after six months, 44.4% showed a >50% seizure reduction, 24.1% including seizure-free, respectively, and 53.7% remained on SGT. Two adverse events were reported, mild skin rash and fever. Focal seizure type presented significantly more positive responses when compared with other seizure types at six months (*p* = 0.0284, Fisher's exact test).* Conclusion*. SGT is an effective treatment with excellent tolerability for drug resistant epilepsy patients. Our data provide evidence that SGT may be used as alternative treatment option when antiepileptic drug does not work in epilepsy children.

## 1. Introduction

Antiepileptic drugs (AEDs) are the most common treatment for epileptic seizures in children. However, approximately one-third of the patients who are given AEDs as the suitable treatment do not achieve optimal seizure control [1]. The patient who has failed to become seizure-free with adequate trials of two AEDs is considered to have drug resistant epilepsy. In particular, severe forms of epilepsy in children such as West syndrome and Lennox-Gastaut syndrome become drug resistant epilepsy despite the use of high doses and several combinations of AEDs. Moreover, the use of AEDs has been associated with various adverse effects, including neurologic toxicity [2]. As a result, there is a constant need for alternative treatment with drug resistant epilepsy.

Complementary and alternative medicine (CAM) is used by patients with various diseases, including epilepsy [3–6]. It has been reported that patients with epilepsy often choose herbal medicine among CAM therapies [7, 8]. Some herbal formulas such as Shihogyejitang (SGT) are often considered as a treatment option of epileptic seizures in the CAM area. SGT, also called chai hu gui zhi tang in Chinese, is a Korean prescription which has been used to treat various disease including epilepsy in traditional Korean medicine. It is composed of nine herbs, Bupleuri radix, Pinelliae tuber, Scutellariae radix, Zizyphi fructus, Ginseng radix, Glycyrrhizae radix, Zingiberis rhizoma, Paeoniae radix, and Cinnamomi cortex. A series of preclinical studies with herbal extracts based on SGT showed that this formula has potential antiepileptic effects [9–15], and clinical studies presented the effect of SGT on reducing seizure frequency with improving cognitive outcome in the patients with epilepsy [16, 17]. However, there have not been enough clinical studies to demonstrate the benefits and adverse effects of SGT for treating epileptic seizures in Korea.

In this study, we conducted a retrospective study aiming to investigate the potential effect and safety of SGT as an alternative treatment for seizure control in children with drug resistant epilepsy.

## 2. Methods

### 2.1. Patient Selection

In this retrospective study, we reviewed the medical records of children under 12 with epilepsy treated with SGT at the Department of Pediatric Neurology, I-Tomato Hospital, Korea, between April 2006 and June 2014. Patients were eligible if they had tried at least two AEDs before starting the treatment with SGT but still had one or more seizure per day. The last follow-up was conducted during November 2014 in order to collect data on the current conditions and the reason for discontinuation of SGT had it occurred. Gachon University Institutional Review Board granted an ethical approval for this study.

Data on the following properties were obtained from medical records: age, gender, seizure type, epilepsy syndromes, initial seizure frequency, AEDs used before starting SGT, developmental status, age at onset of the first seizure, age of staring SGT, and original magnetic resonance imaging (MRI), and EEG data. Classification of seizure types, electroclinical syndromes, and other epilepsies was based on the 2010 ILAE report [21].

### 2.2. Treatment

SGT was administered until the patients achieved seizure-free and maintained the state of remission for over two years. SGT was stopped when seizures increased or unacceptable toxicity occurred. If seizure frequency had not changed for at least two weeks, SGT was also stopped.

The daily dose of SGT used in this study consisted of the following: 7.0 g of Bupleuri radix (*Bupleurum falcatum* L.), 4.0 g of Pinelliae tuber (*Pinellia ternate* (Thunb.) Breit.), 2.0 g of Scutellariae radix (*Scutellaria baicalensis* Georgi), 2.0 g of Zizyphi fructus (*Zizyphus vulgaris* var. spinosus), 2.0 g of Ginseng radix (*Panax ginseng* C.A. Meyer), 1.5 g of Glycyrrhizae radix (*Glycyrrhiza uralensis* Fisch), 1.0 g of Zingiberis rhizoma (*Zingiber officinale* Roscoe), 2.5 g of Paeoniae radix (*Paeonia lactiflora* Pallas), and 2.5 g of Cinnamomi cortex (*Cinnamomum cassia* Blume). The herbal decoction was made in the hospital pharmacy. All the herbs comprising SGT were mixed with 120 mL of purified water and then decocted for 120 minutes at 102°C to 103°C until half of the original amount of liquid was left. 20 mL of the decoction was packed into each vacuum pouch by an automatic packing machine. Patients of age five or older were asked to administer one pack of the decoction 30 minutes after each meal, three times a day. For patients under five, the daily dosage was adjusted based on the conversion table of von Harnack.

The patients were required to make visits to the hospital every two to four weeks. At every visit, data on the patients' seizure frequency and adverse events were collected.

It was attempted to decrease or completely stop AED intake when the patients' >50% seizure reduction level was being sustained at least four weeks.

### 2.3. Assessment of Seizure Frequency

The effect of SGT on seizure control was measured mainly by change in seizure frequency. Seizure frequency was obtained through parental reports and seizure diaries. The average seizure frequency per month was compared to the seizure frequency level prior to beginning the SGT treatment. Seizure control was categorized as follows: (1) seizure-free state; (2) >90% reduction; (3) 50–90% reduction; (4) <50% reduction; (5) no change; and (6) increased seizure frequency.

### 2.4. Endpoints and Statistical Analysis

The primary endpoint of this study was the response rate at six months. The patients who achieved a >50% decrease in seizure frequency compared to the baseline were identified as responders, and response rate was defined as the proportion of the responders.

The secondary endpoints were the seizure-free rate at six months, the retention rate at six months, discontinuation rate of AED intake, and safety. Seizure-free rate was defined as the proportion of the patients who achieved and stayed seizure-free. Retention rate was defined as the proportion of the patients who continued with the SGT treatment. Discontinuation rate of AED intake was defined as the proportion of patients who decreased the number of AEDs including withdraw of AEDs. All patients were evaluated for toxicity, including physical examination and laboratory findings every month during treatment. Laboratory findings included complete blood count with differential count, liver function test, renal function test, and electrolytes.

Statistical analysis was conducted using SPSS (v. 18.0). Two-tailed Fisher's exact test and Mann-Whitney *U* test were applied for analyzing categorical and continuous variables respectively. A *p* value less than 0.05 was considered statistically significant.

## 3. Results

### 3.1. Patient Characteristics

Fifty-four patients were eligible for analysis. Patient demographics are summarized in Table 1. The median age of experiencing the first seizure was 7.5 months (range: 1 day to 6 years), and the median age of beginning the SGT treatment was 16.5 months (range: 1 month to 10.3 years). The median seizure frequency was 8 times/day (range: 1/day to 270/day). As for seizure types, 17 patients were classified as experiencing generalized seizure, 14 were experiencing focal seizure, 21 had epileptic spasms, and the remaining two were unclassified. Nineteen patients had West syndrome and 13 patients had Lenox-Gastaut syndrome. Out of 54 patients, 25 showed abnormalities on brain MRI. Excluding only five of all patients, 49 were diagnosed with delayed mental or physical development. The median number of AEDs that were used but had failed as treatments prior to the beginning of the SGT treatment was 3 (range: 2 to 7). The median number of AEDs that were being used at the beginning of the SGT treatment was 3 (range: 1 to 7). The median duration for the SGT treatment was 6 months (range: 1 to 83 months).

### 3.2. Response Rate and Seizure-Free Rate

The response rate of SGT at six months was 44.4% and the seizure-free rate at six months was 24.1%. The changes of seizure frequency at 1, 3, 6, and 12 months are presented in Table 2.

The changes of seizure frequency according to types of seizure and epilepsy syndrome are presented in Table 3. Patients with focal seizure type presented significantly more positive responses when compared with other seizure types at six months (*p* = 0.0284, Fisher's exact test). Patients with West syndrome and Lennox-Gastaut syndrome showed more favorable outcomes than patients with other epileptic syndromes, but there was no statistical difference.

The effects of clinical parameters in seizure outcomes are shown in Table 4. The median number of AEDs that had been tried before the initiation of the SGT treatment in responders was significantly smaller than that of the nonresponders at three months (*p* = 0.030, Mann-Whitney *U* test).

### 3.3. Retention Rate

The retention rate of SGT at six months was 53.7% (29/54 patients). The retention rates at 1, 3, and 12 months are shown in Table 2.

The main reason for discontinuation was that the guardians of patients felt that herbal medicine as an alternative therapy did not fully meet their expectations (*n* = 21); the guardians of those patients decided to quit the SGT treatment although those patients showed a decrease in seizure frequency during the SGT treatment. 17 of those 21 had a result of >90% reduction of seizure.

Other reasons of discontinuation were ineffectiveness (*n* = 15), financial burdensomeness (*n* = 9), achieving seizure remission for over two years (*n* = 5), adverse events (*n* = 1), and unknown reasons (*n* = 1).

### 3.4. Long-Term Outcomes

There were eleven patients who continued the SGT treatment for over 12 months. Among these patients, five tapered off the use of AEDs during the SGT treatment and were ordered to discontinue the SGT treatment as they maintained the state of remission for over two years. These five patients were all confirmed to be seizure-free without medication other than SGT at the last follow-up. The remaining six patients were maintaining a >90% seizure reduction level until their guardians decided on terminating the treatment with SGT. At the last follow-up conducted after the termination of the SGT treatment, two out of the six reported to have maintained a >90% seizure reduction level whereas the remaining four reported an increased seizure frequency.

### 3.5. AED Usage during the SGT Treatment

The discontinuation rate of AED intake at the last follow-up was 42.6%. In other words, twenty-three patients decreased the number of AEDs including 11 patients who were able to completely withdraw during the SGT treatment. Seventeen out of whom were able to do so with a >90% seizure reduction.

Out of the 54 patients, 29 retained the initial level of AED usage throughout the SGT treatment; 20 of them showed a >50% reduction in seizure frequency.

There were two patients who were prescribed to additional AEDs over the course of the SGT treatment. One of them showed a 50–90% reduction in seizure frequency at one month and a >90% reduction at three months. But seizure frequency at five months had been increased, so valproate was added at five months but it was also not effective. The other patient did not show a response in seizure control with the SGT treatment, but the guardian of the patient strongly wanted to continue the SGT treatment. Therefore, levetiracetam and topiramate had been added after two months of treatment with SGT, but those were also not effective.

### 3.6. Adverse Events

Adverse events were reported in two patients (3.7%). One patient experienced mild skin rash on his trunk within a week of the SGT treatment, but it was diminished within two days without a change in the administration of SGT. One patient experienced mild fever three months after beginning the SGT treatment. Other adverse events except these two were not reported in the physical examination and laboratory testing.

## 4. Discussion

This study evaluated the effect and safety of SGT in children with drug resistant epilepsy. We observed that SGT improved seizure frequency with few adverse events in children with epilepsy who previously had unsuccessful AED trials. Using intention-to-treat analysis, it can be seen that among all patients 44.4% achieved a >50% reduction in seizures including 24.1% seizure-free after six months of the SGT treatment.

Herbal medicines have been widely used in East Asia since ancient times, and some prescriptions of herbal medicine are often regarded as an alternative option for treating epileptic seizures in the field of CAM. In our institution we have different herbal formulas as the treatment options for the patients with epileptic seizures. A herbal prescription suitable for a single individual with epileptic seizures is selected from these options with herbal medicine through the process of the distinct diagnostic method in traditional Korean medicine. SGT is one of the optional formulas for epileptic seizures in our institution. It remains unclear how SGT can make antiepileptic effect. However, several preclinical studies have suggested the mechanism of action of it in which SGT showed inhibitory effects on pentylenetetrazole-induced bursting activities in snail neurons to be characteristic of seizure discharge [10–12] and demonstrated a scavenging activities for free radicals generated within the iron-induced epileptogenic regions of rat brains [15].

There has yet to be a study like this one reporting herbal medicine's effects on children who had not responded to AEDs. Hence, the effect of SGT in this study was assessed by comparing with the results of the studies on the ketogenic diet, a field in which the most research on drug resistant epilepsy exists [22–24]. The results of the three main studies on ketogenic diets with similar patient populations as this study are reported in Table 5 [18–20]. These studies present a range of 26.2–57.8% of patients with a >50% seizure reduction including 13.6–33.2% who became seizure-free at six months. The results of the SGT treatment were comparably favorable to these three studies.

It is worth noting that the ratio of patients who discontinued AED intake was higher in our study than that of the studies on ketogenic diet. The study of Kang et al. reported that 26.1% were able to decrease the number of AEDs, with 6.5% completely withdrawing [18]. In our study, 42.6% were able to decrease the number of AEDs, including 20.4% who completely withdraw. It was attempted in both studies to decrease or completely stop AED intake when the patients' >50% seizure reduction level was being sustained. The patients in our study had chosen herbal medicine as CAM after conventional treatments had failed or resulted in intolerable adverse effects for them. Hence, the high ratio of AED withdrawal during the SGT treatment can be seen as carrying high clinical significance.

Another important consideration with the use of SGT in drug resistant epilepsy is that tolerability and safety are favorable. In our study, two adverse events—drug eruption, fever—were reported. There have been a few reports of drug eruptions with herbal medications containing* Paeonia lactiflora* Pallas, one of the active ingredients in SGT [25, 26]. Although it may have been the cause of the patient's symptom, clinical possibility of SGT causing the fever seems to be low. To our knowledge, fever has not been a reported adverse effect for either SGT or any other prescriptions including the herbs used in making SGT. Other than the patient who discontinued the SGT treatment citing fever as a reason, no patient discontinued the treatment or needed dose modifications due to adverse events. The use of ketogenic diet, which seems to be an effective treatment for drug resistant epilepsy, often accompanies reports on vomiting, diarrhea, kidney stones, growth retardation, food refusal, and so forth, leading to a lower compliance rate [27–29]. Low toxicity and excellent tolerability of SGT qualifies it as a suitable candidate for drug resistant epilepsy in children.

Retention rates in our study are similar to those from the reports on ketogenic diet despite the fact that far more adverse events had been associated with ketogenic diet compared to the SGT treatment (Table 5). In our study, guardians of 21 patients who had discontinued the treatment despite achieving seizure reduction indicated the SGT treatment could not meet their expectations and wanted to try other CAM that might result in complete remission of seizure. Several explanations have been suggested. One possibility is that the patients who choose herbal medicine as CAM have tendencies to have exaggerated expectations, being without the knowledge of its practical effectiveness. This is because of the lack of research that evaluated the effects of herbal medicine. More research in the future should be able to compensate for such shortfall. Another possible explanation is that the guardians of 21 patients may have considered the “cost-effectiveness” of SGT. In Korea, herbal decoctions can be financially burdensome as they are not subject to medical insurance. However, some medications can be prepared in forms of extracts, which can be insurance-coverable. Hence, the cost problem could be mitigated.

The limitation of our study is that it was conducted at a single institution with a single treatment arm. It was also a retrospective review, rather than a controlled experiment, including only 54 patients. Moreover, the lack of similar studies involving SGT led to using studies on ketogenic diet, for which the effectiveness on drug resistant epilepsy was established, for comparison. Comparisons with the effect of ketogenic diet may be biased by differences in patient selection.

## 5. Conclusion

This study evaluated the effect and safety of SGT for drug resistant childhood epilepsy. We observed that SGT decreased seizure frequency with low toxicity. Therefore, SGT shows a potential for seizure management in children with drug resistant epilepsy. Even though this study has a limitation of not-randomized design with small population, this is the first retrospective study evaluating the efficacy and safety of herbal medicine for epilepsy children.

Based on this study, a large prospective study with a control group should be considered to evaluate the efficacy of SGT.

## Figures and Tables

**Table 1:** Baseline characteristics of the subjects (*N* = 54).

	Number of patients (%)
Gender	
Male	31 (57.4)
Female	23 (42.6)
Seizure frequency	
1–10 times/day	33 (61.1)
11–50 times/day	13 (24.1)
>51 times/day	8 (14.8)
Seizure type	
Generalized^†^	17 (31.5)
Tonic-clonic	2 (3.7)
Atypical absence	6 (11.1)
Myoclonic	1 (1.9)
Tonic	12 (22.2)
Atonic	5 (9.3)
Focal	14 (25.9)
Epileptic spasms	21 (38.9)
Unclassified	2 (3.7)
Electroclinical syndrome and other epilepsies	
Neonatal epileptic encephalopathy	2 (3.7)
Epilepsy of infancy with migrating focal seizures	1 (1.9)
West syndrome	19 (35.2)
Lenox-Gastaut syndrome	13 (24.1)
Generalized epilepsy without a known structural causes	3 (5.6)
Focal epilepsy	
With a known structural or metabolic causes	4 (7.4)
Without a known structural or metabolic causes	9 (16.7)
Epilepsies of unknown causes	3 (5.6)
Age at the first seizure	
Median (range)	7.5 months (1 day to 6 years)
Age at the start of the SGT	
Median (range)	16.5 months (1 month to 10.3 years)
Brain MRI at starting SGT	
Normal	29 (53.7)
Abnormal	25 (46.3)
Developmental status	
Delayed	49 (90.7)
Normal	5 (9.3)
Number of previously used AEDs	
2	17 (31.5)
3	18 (33.3)
4	12 (22.2)
5	4 (7.4)
>6	3 (5.6)
Previously used AEDs	
Sodium valproate	25 (46.3)
Vigabatrin	24 (44.4)
Levetiracetam	23 (42.6)
Topiramate	22 (40.7)
Phenobarbital	14 (25.9)
Clobazam	12 (22.2)
Lamotrigine	8 (14.8)
Oxcarbazepine	8 (14.8)
Clonazepam	7 (13)
Divalproex sodium	5 (9.3)
Rufinamide	4 (7.4)
Zonisamide	4 (7.4)
Phenytoin	3 (5.6)
Carbamazepine	2 (3.7)
Ethosuximide	1 (1.9)
Lacosamide	1 (1.9)
Number of concomitant AEDs at start of herbal medicine	
1	1 (1.9)
2	21 (38.9)
3	15 (27.8)
4	13 (24.1)
5	3 (5.6)
>6	1 (1.9)

SGT, Shihogyejitang; MRI, magnetic resonance imaging; AED, antiepileptic drug.

^†^5 patients had mixed seizure type.

**Table 2:** Seizure outcomes and retention rates at 1, 3, 6, and 12 months.

	1 month	3 months	6 months	12 months
Responders				
Seizure-free	10	19	13	8
>90% reduction	15	11	10	4
50–90% reduction	13	7	1	0
Nonresponders				
<50% reduction	11	2	1	0
Not changed	3	4	2	0
Increased	2	1	2	0
Response rate	70.4% (38/54)	68.5% (37/54)	44.4% (24/54)	22.2% (12/54)
Retention rate	90.7% (49/54)	81.5% (44/54)	53.7% (29/54)	22.2% (12/54)

**Table 3:** Seizure outcomes according to seizure types and epileptic syndromes at six months.

	Seizure types			Epileptic syndromes	
	Generalized	Focal	Epileptic spasms	West syndrome	LGS
Number of patients	9	11	9	8	7
Responders					
Seizure-free	3	4	6	6	2
>90% reduction	3	5	2	1	4
50–90% reduction	0	1	0	0	0
Nonresponders					
<50% reduction	1	0	0	0	0
Not changed	1	0	1	1	1
Increased	1	1	0	0	0
Response rate	66.7% (6/9)	90.9% (10/11)	88.9% (8/9)	87.5% (7/8)	85.7% (6/7)

LGS, Lenox-Gastaut syndrome.

**Table 4:** Effects of clinical parameters on seizure outcomes.

		3 months	6 months	12 months
Age at the onset of first seizure (months)	Responders	13.47 ± 16.85	16.96 ± 19.66	18.92 ± 17.57
	Nonresponders	8.69 ± 7.27	7.71 ± 6.83	9.97 ± 13.24
	*p* value	0.615	0.146	0.051

Age at the start of the SGT (months)	Responders	35.81 ± 34.77	43.58 ± 38.22	40.42 ± 31.54
	Nonresponders	28.71 ± 26.23	24.07 ± 23.35	31.62 ± 32.56
	*p* value	0.776	0.086	0.139

Treatment duration (months)	Responders	22.34 ± 26.37	26.62 ± 29.16	21.50 ± 17.66
	Nonresponders	20.02 ± 26.39	16.35 ± 22.54	21.65 ± 28.29
	*p* value	0.970	0.356	0.382

Number of AEDs that had been tried	Responders	3.00 ± 1.05	3.05 ± 1.16	2.92 ± 1.00
	Nonresponders	3.71 ± 1.21	3.38 ± 1.15	3.31 ± 1.18
	*p* value	0.030^*∗*^	0.228	0.313

Data are presented as the mean ± SD. Mann-Whitney *U* tests were used for comparison between responders and nonresponders. ^*∗*^Statistically significant at *p* < 0.05.

SGT, Shihogyejitang; AED, antiepileptic drug.

**Table 5:** Comparison to studies on ketogenic diet in patients with refractory epilepsy.

	Treatment	Number	Median ageat start of treatment	Response rate						Retention rate		
				At 3 months		At 6 months		At 12 months		At 3 months	At 6 months	At 12 months
				>50% reduction^††^	Seizure-free	>50% reduction^††^	Seizure-free	>50% reduction^††^	Seizure-free			
Our study	SGT	54	16.5 months	68.5%	35.2%	44.4%	24.1%	22.2%	14.8%	81.5%	53.7%	22.2%
Kang et al., 2005 [18]	Ketogenic diet	199	57.9 months^†^	61.8%	35.2%	57.8%	33.2%	41.2%	25.1%	87.9%	68.3%	45.7%
Sharma et al., 2009 [19]	Ketogenic diet	27	2.5 years	59.3%	11.1%	48.1%	14.8%	37.0%	18.5%	88.9%	55.6%	37.0%
Suo et al., 2013 [20]	Ketogenic diet	317	39.6 months^†^	35.0%	20.8%	26.2%	13.6%	18.6%	10.7%	62.8%	42.0%	24.3%

^†^Mean age.

^††^ >50% reduction included seizure-free cases.

SGT, Shihogyejitang.
